# Transient bilateral abducens neuropathy with post-tetanic facilitation and acute hypokalemia associated with oxaliplatin: a case report

**DOI:** 10.1186/1752-1947-4-36

**Published:** 2010-02-02

**Authors:** Min-Han Tan, Wen Yee Chay, Jia Hui Ng, Bin Tean Teh, Lita Chew

**Affiliations:** 1 Department of Medical Oncology, National Cancer Centre, 11 Hospital Drive, Singapore 169610, Republic of Singapore; 2 NCCS-VARI Laboratory of Translational Cancer Research, National Cancer Centre, 11 Hospital Drive, Singapore 169610, Republic of Singapore; 3 Oncology Pharmacy, National Cancer Centre, 11 Hospital Drive, Singapore 169610, Republic of Singapore

## Abstract

**Introduction:**

Oxaliplatin is a cytotoxic platinum compound that is in widespread use in the treatment of gastrointestinal cancers. It has been occasionally associated with acute motor neuropathy, but the precise mechanism is uncertain. To the best of our knowledge, we report the first case of a patient demonstrating post-tetanic facilitation in the setting of transient bilateral abducens neuropathy and hypokalemia, after being infused with oxaliplatin.

**Case presentation:**

A 47-year-old Indian woman with metastatic gastric cancer was receiving an oxaliplatin infusion at the initiation of her third cycle of palliative chemotherapy. She developed acute bilateral abducens neuropathy with post-tetanic facilitation alongside acute laryngopharyngodysesthesia and hypokalemia. Following supportive management, including potassium infusion and warming, her neurological signs and symptoms were spontaneously resolved. This syndrome did not recur in subsequent cycles following prolongation of infusion duration and the addition of supportive calcium and magnesium infusions.

**Conclusion:**

The novel clinical observation of post-tetanic facilitation highlights a possible involvement of voltage-gated channels at the presynaptic terminals in the mechanism of acute oxaliplatin neurotoxicity.

## Introduction

Oxaliplatin is a recently developed cytotoxic platinum compound that is in widespread use, particularly for the treatment of gastrointestinal cancers [1]. The association between oxaliplatin and acute and chronic sensory neuropathy is well recognized, but acute motor neuropathy is also reported, albeit much less frequently [2]. The exact mechanisms of both acute and chronic neurotoxicity remain uncertain, despite extensive clinical experience with the compound. It is conventionally regarded that the voltage-gated sodium channels are involved in mechanisms of acute neurotoxicity [3], possibly through a pathway involving calcium ions [4]. The use of calcium and magnesium infusions is currently under investigation [5].

## Case presentation

We report a novel observation in a 47-year-old Indian woman with no comorbidities who developed acute bilateral abducens neuropathy with post-tetanic facilitation alongside acute hypokalemia after infusion of oxaliplatin. She was first diagnosed with peritoneal carcinomatosis arising from metastatic gastric adenocarcinoma, and was commenced on a palliative chemotherapy regimen of continuous infusion 5-fluorouracil (5-FU) 200 mg/m^2^/day and weekly oxaliplatin (50 mg absolute dose). After the first cycle totaling 150 mg oxaliplatin, during which significant clinical and radiological improvement in her intestinal function was documented permitting administration of oral medication, her regimen was modified to a three-weekly regimen of oxaliplatin (130 mg/m^2 ^on day 1 over 2 hours) and capecitabine (2000 mg/m^2^/day on day 1 evening - day 15 morning) (XELOX). One hour into the infusion of oxaliplatin of the 2nd cycle of XELOX, she developed acute neuropathy comprising acute bilateral abducens neuropathy, dysarthria, laryngopharyngodysesthesia, perioral and peripheral numbness. In addition to oxaliplatin, she had received 8 mg of intravenous dexamethasone and 8 mg of intravenous ondansetron as premedication. The patient had not taken any oral medications other than capecitabine up to one week previously. The patient demonstrated physical signs consistent with bilateral abducens neuropathy, with bilateral gaze-evoked diplopia. Surprisingly, post-tetanic facilitation was observed, with unilateral resolution of diplopia and abducens neuropathy following sustained lateral gaze over one minute. Contralateral abducens neuropathy remained unchanged following apparent resolution of the unilateral abducens neuropathy. The apparently resolved unilateral abducens neuropathy recurred following a two-minute rest. Decreased tendon reflexes diffusely were noted, but no focal or generalized weakness was found. New electrocardiographic changes in terms of diffuse T-wave inversions were noted, as well as mild elevation of her serum creatine kinase-MB (CK-MB) levels by mass assay. Also noted was a serial decrease over 16 hours (7.0 μg/L immediately, 5.2 μg/L 8 hours later and 3.9 μg/L 16 hours later), but the diffuse T-wave inversions persisted. Corresponding serial serum creatine kinase (CK) levels were 72 μg/L, 63 μg/L and 52 μg/L, and troponin-T levels were not elevated at any of these three-time points.

Baseline blood counts and electrolytes drawn two days prior were unremarkable; in particular, her potassium level was 3.5 mmol L^-1 ^(3.3-4.9 mmol L^-1^). She was asymptomatic up till this event. In particular, there was no intervening symptom of vomiting or diarrhoea, and the patient did not report the use of any oral medications. The patient did not have prior hypokalemia up to this point. Electrolytes drawn immediately upon onset of neurological symptoms revealed acute hypokalemia, with a serum potassium level of 2.5 mmol L^-1 ^with a normal serum bicarbonate level of 23.1 mmol L^-1 ^(19.0-31.0 mmol L^-1^), normal corrected serum total calcium level of 2.28 (2.10-2.60 mmol L^-1^) and magnesium level of 0.78 mmol/L (0.70-0.95 mmol L^-1^).

The patient was placed in a warm environment and an aggressive potassium replacement was undertaken. She did not receive calcium or magnesium infusions. The bilateral abducens neuropathy, dysarthria and laryngopharyngodysesthesia resolved over two hours. The hypokalemia, perioral and peripheral numbness resolved over twelve hours. Due to the short duration of the episode, no electromyogram (EMG) or nerve conduction testing was performed. The diffuse T-wave inversions on ECG did not resolve and remained six months following this event.

The patient was re-challenged with the same dose of oxaliplatin for her fourth cycle, with infusion time extended from 2 to 6 hours, together with calcium and magnesium infusions, and did not experience subsequent neurotoxicity. She continued to receive second-line irinotecan and cisplatin, third-line docetaxel, fourth-line epirubicin, cisplatin and 5-fluorouracil and fifth line 5-fluorouracil and cetuximab. All treatments were tolerated reasonably without further adverse events. The patient eventually passed away from progressive disease approximately fifteen months after initial diagnosis while on best supportive care.

## Discussion

While the mechanism of oxaliplatin neurotoxicity remains uncertain, EMG studies have demonstrated peripheral motor nerve hyperexcitability coinciding with symptoms of acute neuropathy, including cold-induced paresthesias and larygopharyngodysesthesias [6]. It is conventionally regarded that voltage-gated sodium channels are involved. Preclinical studies showed that oxaliplatin causes prolonged opening of voltage-gated sodium channels in sensory nerves with a resulting hyperexcitable state [7]. It has been suggested in preclinical studies that oxaliplatin may mediate this channelopathy through the rapid chelation of unbound calcium through its oxalate metabolite [4].

A previous ex vivo study investigating voltage-gated potassium channels using blockers such as apamin has been performed, with no apparent correlation between phenotypes induced by apamin and acute oxaliplatin neurotoxicity [8]. The authors, however, argue that a population of apamin-resistant, calcium-dependent potassium channels has been identified, and that calcium chelation by oxalate provides a viable mechanism for this to occur. Indeed, recently reported ex vivo work suggests that oxaliplatin may interfere with voltage-gated potassium channels [9,10]. Unexpectedly, one of these two reports did not detect an effect of oxaliplatin on voltage-gated sodium channels [10].

A single case report previously has reported an association between oxaliplatin and bilateral abducens nerve palsy in a patient heavily exposed to previous cisplatin [11], but did not report on the phenomenon of post-tetanic facilitation or associated hypokalemia. Post-tetanic facilitation has not been previously recognized, and may imply the involvement of voltage-gated channels in the presynaptic terminal, where both potassium and calcium channels are present. Calcium-activated presynaptic potassium channels in Xenopus are known to regulate transmitter release magnitude during single action potentials by altering the rate of action potential repolarization, and thus the magnitude of peak calcium current [12].

The acute hypokalemia we observe has been reported in a separate case report where a coma associated with hypokalemia and hypocalcemia was reported after infusion of oxaliplatin [13]. The mechanism is uncertain. Given that our patient presented with hypokalemia but not hypocalcemia, the data does not support systemic calcium chelation as the primary mechanism of acute oxaliplatin-induced neurotoxicity, but it is conceivable that a specific locations along the peripheral nerve may be more vulnerable to accumulation of oxaliplatin or its metabolites [1]. For example, platinum has been shown to accumulate in dorsal root ganglia in rodents administered oxaliplatin.

We considered the possibility of the acute hypokalemia causing the post-tetanic facilitation that we observed. Acute hypokalemia is associated with axonal and muscle membrane hyperpolarization [14,15]. It must be acknowledged that although our review of the literature did not report a previous association between hypokalemia and post-tetanic facilitation, the mechanisms of hypokalemia in inducing weakness are complex and as-yet poorly understood. Hence, a possible contribution of hypokalemia to post-tetanic facilitation cannot be definitively excluded. However, recent electrophysiologic investigations of patients with acute hypokalemia highlight its contribution to axonal hyperpolarization, with a resulting activity-dependent conduction block worsening, rather than improving weakness [14]. Hence, this data suggests that hypokalemia is not the primary mechanism for post-tetanic facilitation.

## Conclusion

In summary, while the mechanism of acute oxaliplatin-induced neuropathy remains uncertain, our novel clinical observation of post-tetanic facilitation alongside acute hypokalemia highlights voltage-gated channels at the presynaptic nerve terminal for investigation in the mechanism of acute oxaliplatin neurotoxicity.

## Consent

Written informed consent was obtained from the patient's family for publication of this case report. A copy of the written consent is available for review by the Editor-in-Chief of this journal.

## Competing interests

The authors declare that they have no competing interests.

## Authors' contributions

MHT wrote the manuscript. JHN and LC obtained data and reviewed the literature. CWY and BTT helped write the manuscript. All authors read and approved the final manuscript.
